# Effects of the cardiac myosin activator Omecamtiv-mecarbil on severe chronic aortic regurgitation in Wistar rats

**DOI:** 10.1186/s12872-018-0831-3

**Published:** 2018-05-21

**Authors:** Bachar El-Oumeiri, Kathleen Mc Entee, Filippo Annoni, Antoine Herpain, Frédéric Vanden Eynden, Pascal Jespers, Guido Van Nooten, Philippe van de Borne

**Affiliations:** 1 Department of Cardiac surgery, Erasme Hospital, ULB, 808 Lennik road, 1070, Brussels, Belgium; 2 Laboratory of Physiology and Pharmacology, ULB, Brussels, Belgium; 3 Department of intensive care, Erasme Hospital, ULB, Brussels, Belgium; 4 Department of Cardiology, Erasme Hospital, ULB, Brussels, Belgium

**Keywords:** Omecamtiv-mecarbil, Aortic regurgitation, Doppler-echocardiography, Wistar rat, Left ventricle

## Abstract

**Background:**

Aortic regurgitation (AR) is a valvular disease that can lead to systolic heart failure. Treatment options besides cardiac surgery are limited and consequently severe AR is associated with higher mortality and morbidity when not operated. In this investigation, we examined the effects of a novel cardiac myosin activator, Omecamtiv-mecarbil (OM), in rats with chronic severe AR.

**Methods:**

AR was created by retrograde puncture of the aortic valve leaflets in 20 adults Wistar rats. 12 animals survived the acute AR phase and were randomized 2 months thereafter into OM (*n* = 7) or placebo groups (*n* = 5). Two rats underwent a sham operation and served as controls. Equal volumes of OM or placebo (NaCl 0.9%) were perfused in the femoral vein by continuous infusion (1.2 mg/kg/hour) during 30 min. Doppler-echocardiography was performed before and at the end of the infusion periods.

**Results:**

OM increased indices of global cardiac function (cardiac output, stroke volume), and increased systolic performance (fractional shortening, ejection fraction, left ventricular end systolic diameter) (all *p* < 0.05). These effects concurred with decreases in indices of LV preload (left atrial size, left ventricular end diastolic diameter) as well in the aortic pre-ejection period / left ventricular ejection time ratio (all *p* < 0.05). The severity score of the regurgitant AR jet did not change. Placebo infusion did not affect these parameters.

**Conclusion:**

The cardiac myosin activator OM exerts favorable hemodynamic effects in rats with experimental chronic AR.

## Background

Aortic regurgitation (AR) is a valvular disease that affects men more than women, and whose incidence increases with age [1]. Severe AR is associated with higher morbidity and mortality compared to the general population [2]. Chronic AR secondary to rheumatic fever is a frequent condition in developing countries and in populations having no adequate access to health care [3]. Chronic severe AR imposes a combined left ventricular (LV) volume and pressure overload. Volume increase is a direct consequence of the regurgitant volume itself, while pressure overload results from increased parietal stress and systolic hypertension [4]. AR is associated with a long asymptomatic period during which the LV progressively enlarges and hypertrophies in response to a chronic volume overload. The increased wall stress and LV volume/mass ratio can lead to impaired LV systolic function, clinical signs of heart failure and, finally, become irreversible and lethal [5]. So far, vasodilators are the only drugs indicated in asymptomatic AR, but their hemodynamic effects are inconsistent and their impact on clinical outcomes is largely uncertain [6, 7]. Rats are convenient animals to evaluate the response of the LV to severe AR as they develop LV abnormalities in a relatively short period of time (weeks). This is in contrast to humans, who can tolerate this condition without apparent LV dysfunction for decades [8]. The rats develop progressive LV dilatation and eccentric hypertrophy, due to a chronic LV volume overload, as well as progressive irreversible LV systolic dysfunction, mimicking closely the evolution of the disease over a much larger time span in humans [9].

In chronic AR, indices of systolic function such as left ventricular ejection fraction (LVEF) are better prognostic indicators than indices of cardiac overload [2]. Moreover, markers of systolic function are useful for decision of a timely surgical valve replacement [10, 11]. Conventional medical treatment of congestive heart failure with altered ejection fraction is based on neuro-hormonal blockade, neuro-hormonal activation being considered responsible for aggravation of heart failure and loss of myocardial contractility [12]. Because of safety issues, conventional therapies that directly target cardiac contractility are sparsely used [13]. Heart failure (HF) remains a major public health problem worldwide. Existing drugs increase cardiac contractility indirectly through signaling cascades but are limited by their mechanism related adverse effects. To avoid this limitation Omecamtiv-mecarbil (OM) was developed. Omecamtiv-mecarbil, formerly called CK-1827452 (Cytokinetics Inc., San Francisco, CA, USA) is a novel drug which improves cardiac contractility by means of cardiac myosin activation. OM accelerated the transition of myosin from the weakly actin-bound to strongly actin-bound state measured by release of Hydrolazed Phosphate (Pi). OM appeared to shift the equilibrium towards myosin adenosine triphosphate (ATP) hydrolysis without affecting the rate of hydrolysis, in addition to accelerating the rate of Pi release. OM decreased the rate of Pi release when actin was removed. This decrease in actin-independent ATP hydrolysis potentially increases the overall energetic efficiency of the system by diminishing ATP use not associated with mechanical work [14]. Consequently, OM increases systolic ejection duration without changing the rate of left ventricular pressure development [15, 16].

In two different canine models of pacing-induced systolic heart failure (after myocardial infarction [15] and in the presence of left ventricular hypertrophy [15]), OM increased systolic wall thickness and fractional shortening (FS), leading to an improved global cardiac function and lowered heart rate (HR), while myocardial energetics and loading conditions did not change. Cardiac morphology alterations in tachycardia-induced cardiomyopathy include chamber dilatation and normal or reduced ventricular wall thickness, with little or no change in myocardial mass. These changes, together with myocardial energy depletion and impaired energy utilization, are reversible once HR normalizes [17].These characteristics differ completely from the chronic volume and pressure overload in severe AR, leading to LV dilatation and eccentric hypertrophy, an ultimately, to an irremediably compromised LV function. Favorable effects of OM in experimental pacing-induced cardiomyopathy may thus not apply to chronic severe AR. Consequently, the goal of the present study was to test the hypothesis that cardiac myosin activation with OM improved left ventricular function in a rat-model of chronic severe aortic regurgitation. The expanded goal of this study was to determine if OM affected AR severity.

## Methods

### Animals

Experiments were approved by the Institutional Animal Care and Use Committee of the Free University of Brussels. Studies were conducted in accordance with the Guide for the Care and Use of Laboratory Animals published by the National Institutes of Health (NIH Publication No. 85–23, revised 1996). Twenty-four male adult Wistar rats (401 ± 90 g body weight) were randomized to a sham intervention (*n* = 4) or to AR creation (*n* = 20). Rats that survived the acute phase (*n* = 12) were randomized into an OM group (*n* = 7) or a placebo group (*n* = 5). The 4 rats (two in the OM group and two in the placebo group) who underwent a sham operation served as controls for the effect of time and measurement repetition on the parameters investigated in the study.

### Interventions

AR was created by retrograde puncture of the aortic valve leaflets under general anesthesia, as previously described [18]. Briefly, the animals were anesthetized with an intraperitoneal injection of 75 mg/kg of ketamine and 0.25 mg/kg of medetomidine. HR and rhythm were monitored via limb leads throughout the procedure. The right internal carotid artery was surgically exposed. A fixed core wire guide .025″ (COOK incorporated, IN, 47404, USA) was advanced toward the aortic valve in a retrograde manner to tear valve leaflets and induce AR. The following echocardiographic criteria with popping sensation at the time of surgery were used to include animals in the study: a jet extent above 30% of the length of the LV and color-Doppler ratio of regurgitant jet width to LV outflow tract diameter above 50% [19]. The 2 sham-operated animals had their right carotid artery cannulated without puncturing the aortic valve. Animals were closely observed during the first hours and days after surgery for any sign of respiratory distress suggestive of acute heart failure.

### Measurements

Transthoracic 2D, M-mode and Doppler echocardiography were performed under general anesthesia with an ultrasound unit (Vivid-7, GE Healthcare, US) equipped with a 10Mhz surgical transducer. Rats were placed in the right and left lateral recumbent positions and their electrocardiogram was monitored via limb leads throughout the procedure. All measurements were made according to the recommendations of the American Society of Echocardiography currently applied to humans [19]. Standard right parasternal (long and short axis) and left apical parasternal views were used for data acquisition. Left atrial size was assessed in right parasternal short axis at the level of the aorta. Diastolic (d) and systolic (s), septal wall thickness (SWT), posterior wall thickness (PWT) and LV diameters (LVEDD, LVESD) were measured in M-mode from a LV short axis view at the level of chordae tendinae and fractional shortening (FS) was calculated. Ejection fraction (EF) were derived using the Teicholz formula. Left ventricle mass was calculated using the American Society of Echocardiography recommended formula: LV mass = 0.8 x {1.04[(LVEDD +PWTd + SWTd)^3^-(LVEDD)^3^]} + 0.6 g. Aortic diameter was measured from the right long axis parasternal view. Aortic flow was measured from the left apical view to calculate forward stroke volume (SV) and cardiac output and to measure pre-ejection period (PEP: delay from Q wave of QRS to aortic opening, ms), LV ejection time (LVET: interval from beginning to termination of aortic flow, ms), and inter-beat interval (RR). Systolic time was determined as PEP + LVET (ms). Diastolic time (ms) consists in RR interval (ms) - systolic time (ms). PEP/LVET ratio was also calculated. PEP/LVET is a more useful index of overall LV performance [20]. This ratio is better correlated with other LV performance measurements than either PEP or LVET, and is considered independent of HR [21]. Severity of the regurgitant aortic jet was subjectively graded (1 to 4).

### Experimental design

Doppler-echocardiography was performed before AR creation, during surgery to confirm the presence and the severity of AR, and 2 months thereafter, both before and after OM (1.2 mg/kg/hour) or placebo (NaCl 0.9%) infusion for 30 min, by means of a femoral vein perfusion. All animals received equal volumes (12 ml/kg) of placebo or OM. This achieved plasma concentration of nearly 400 ng of OM/ml in a previous study [22]. Doppler-Echocardiography was performed after 30 min infusion. All animals remained alive during these experimental sessions which could thus be completed in 5 rats with placebo and 7 rats with OM.

### Statistical analysis

Results are expressed as means ± SD. A 2-factor ANOVA for repeated measures followed by post-hoc Bonferroni corrections for multiple comparisons was used to assess the effects of OM versus placebo, and any interaction between them, after 2 months of AR on the 16 animals. All other statistical analysis consisted of paired-t tests between variables. Significance was set at a *p* value less than 0.05. (SPSS 23.0, IBM, Chicago, Ill, USA).

## Results

### AR and LV measurements (Fig. 1)

AR was achieved in all 20 animals and confirmed by the presence of a regurgitant jet quantified as severe in all animals. Eight animals died of congestive heart failure within 2 months and were not included in the final analysis. After 2 months AR (graduated from 0 to 4) was achieved at 3.67 ± 0.44, and echocardiographic signs of volume overload and eccentric hypertrophy were present with increased left atrial diameter, LVEDD, LVEDV and LV mass (*n* = 12, all *p* < 0.05, paired t tests). Load dependent indices of LV systolic function (FS and EF) were unchanged but LVESD were increased. SV and cardiac output were decreased (*n* = 12, both *p* < 0.01, paired t tests). As expected, no AR was detected in sham operated rats (*n* = 4) with no modifications of LV function or dimension in the placebo group while only FS and EF increased after injection of OM (*p* = 0.011 and *p* = 0.032, respectively) (Table 1).

**Fig. 1:**
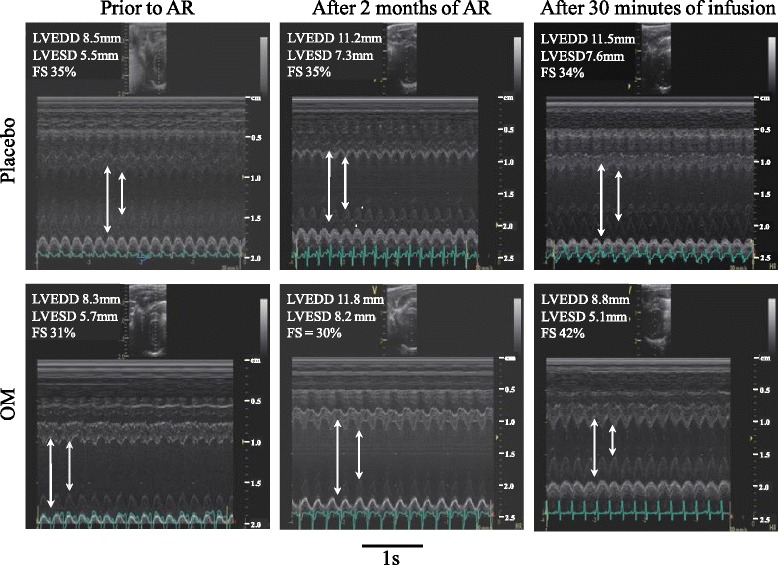
Illustrative examples of M-mode echocardiography recordings in 2 Wistar rats during the entire study. The figure displays left ventricular end diastolic diameter (LVEDD, mm) and fractional shortening (FS, %) prior to AR (left tracings), after 2 months of AR (middle tracings) and after 30 min of placebo (upper tracings) and OM infusions (lower tracings)

**Table 1:** Two-way ANOVA statistics with Bonferroni correction at base (T1), before injection (T2) and after injection (T3) for all sham animals (*n* = 4)

	T1	T2	*p*	T3	*p*
Heart Rate (beats/min)	258 ± 11	270 ± 7	0,756	193 ± 15	0,251
Left Atrium (mm)	4.6 ± 0.2	6.2 ± 0.3	0,872	5.5 ± 0.2	0,118
LVEDD (mm)	7.9 ± 0.5	11.1 ± 0.4	0,938	10.1 ± 0.5	0,209
LVESD (mm)	5.5 ± 0.4	7.6 ± 0.3	0,736	6.1 ± 0.4	0,072
FS (%)	31.5 ± 1.4	30.5 ± 1.0	0,937	35.4 ± 1.5	0,011
EF (%)	64.1 ± 2.0	63.9 ± 1.4	0,630	68.8 ± 2.5	0,032
Stroke Volume (ml)	0.34 ± 0.03	0.22 ± 0.18	0,448	0.30 ± 0.04	0,762
Cardiac Output (ml/min)	79.5 ± 9.3	52.0 ± 4.5	0,890	61.6 ± 8.6	0,393
SWTs (mm)	2.1 ± 0.1	2.3 ± 0.2	0,671	2.7 ± 0.2	0,457
SWTd (mm)	1.4 ± 0.1	1.7 ± 0.2	0,971	2.1 ± 0.2	0,092
Systolic time (ms)	126 ± 3.7	134 ± 2.6	0,245	135 ± 2.6	0,153
Diastolic time (ms)	139 ± 8.4	135 ± 8.3	0,207	157 ± 14.5	0,138
PEP (ms)	15.0 ± 1.6	25.4 ± 1.3	0,413	21.8 ± 1.0	0,308
LVET (ms)	110 ± 4.4	109 ± 2.8	0,310	113 ± 3.9	0,268
PEP/LVET	0.13 + 0.02	0.23 + 0.02	0,800	0.19 + 0.01	0,968
Systolic time/RR	0.48 + 0.02	0.50 + 0.02	0,344	0.47 + 0.02	0,370

Values are mean ± SD. *LVEDD* Left ventricle end-diastolic diameter, *LVEDV* Left ventricle end-diastolic volume, *LVESD* Left ventricle end-systolic diameter, *LVESD* Left ventricle end-systolic volume, *FS* Fractional shortening, *EF* Ejection fraction, *SV* stroke volume, *SWTs* septal wall thickness at end-systole, *SWTd* septal wall thickness at end-diastole, *PEP* aortic pre-ejection period, *LVET* Left ventricular ejection time, *RR* inter-beat interval

### Effects of placebo in rats with AR (Table 2)

Before infusion, there was no difference in echocardiographic results between the 2 groups (placebo versus OM). NaCl infusion affected none of the echocardiographic parameters of global and systolic cardiac function neither the indices of LV preload (*n* = 5, *p* > 0.06, paired t tests).

**Table 2:** Two-tailed T-test before and after Placebo infusion on LV function after 2 months of AR in a rat model (*n* = 5)

	Before infusion	After infusion	*p*-value
Heart Rate (beats/min)	249 ± 18	220 ± 41	0.437
Left Atrium (mm)	6.2 ± 0.8	5.8 ± 1.1	0.541
LVEDD (mm)	10.6 ± 0.8	12.24 ± 1.07	0.092
LVESD (mm)	7.5 ± 0.6	6.9 ± 0.84	0.341
FS (%)	28.8 ± 1.4	29.6 ± 5.2	0.706
EF (%)	60.8 ± 1.8	61.0 ± 8.4	0.968
Stroke Volume (ml)	0.24 ± 0.04	0.24 + 0.08	0.890
Cardiac Output (ml/min)	60 ± 8	60 ± 12	0.369
SWTs (mm)	3.0 ± 0.16	2.9 ± 0.47	0.122
SWTd (mm)	1.6 ± 0.17	1.7 ± 0.29	0.281
Systolic time (ms)	136 ± 5	123 ± 17	0.340
Diastolic time (ms)	144 ± 24	173 ± 41	0.054
PEP (ms)	25.8 ± 4.6	21.2 ± 6.2	0.125
LVET (ms)	110 ± 8	102 ± 14	0.490
PEP/LVET	0.24 ± 0.05	0.20 ± 0.05	0.182
Systolic time/RR	0.49 ± 0.05	0.42 ± 0.05	0.047

Values are mean ± SD. Left ventricle end-diastolic diameter; LVEDV: Left ventricle end-diastolic volume; LVESD: Left ventricle end-systolic diameter; LVESD: Left ventricle end-systolic volume; FS: Fractional shortening; EF: Ejection fraction; SV: stroke volume; SWTs: septal wall thickness at end-systole; SWTd: septal wall thickness at end-diastole PEP: aortic pre-ejection period; LVET: Left ventricular ejection time; RR: inter-beat interval

### Effects of OM in rats with AR (Table 3)

OM increased indices of global cardiac function (SV, cardiac output), decreased HR and increased systolic performance (FS, EF) (*n* = 7, all *p* < 0.05, paired t tests). These effects concurred with decreases in measures of LV preload (Left atrial diameter, LVEDD), and a decreased PEP/LVET ratio (*n* = 7, all *p* < 0.05, paired t tests). OM did not affect the severity score of the AR jet.

**Table 3:** Two-tailed T-test before and after OM infusion on LV function after 2 months of AR in a rat model (*n* = 7)

	Before infusion	After infusion	*p*-value
Heart Rate (beats/min)	253 ± 22	207 ± 35	0.091
Left Atrium (mm)	6.2 ± 0.8	5.5 ± 0.7	0.037
LVEDD (mm)	11.6 ± 1.09	9.0 ± 1.51	0.003
LVESD (mm)	7.8 ± 0.91	5.4 ± 1.30	0.009
FS (%)	32.1 ± 3.4	41.1 ± 5.3	0.004
EF (%)	65.0 ± 4.7	76.6 ± 5.8	0.002
Stroke Volume (ml)	0.19 ± 0.06	0.36 + 0.11	0.011
Cardiac Output (ml/min)	44 ± 16	76 ± 14	0.027
SWTs (mm)	2.67± 0.48	3.33 ± 0.51	0.390
SWTd (mm)	1.6 ± 0.17	2.4 ± 0.39	0.274
Systolic time (ms)	133 ± 7	147 ± 8	0.003
Diastolic time (ms)	127 ± 16	141 ± 33	0.384
PEP (ms)	25.0 ± 2.3	22.4 ± 1.8	0.042
LVET (ms)	108 ± 6	125 ± 9	0.002
PEP/LVET	0.23 ± 0.02	0.18 ± 0.02	0.007
Systolic time/RR	0.51 ± 0.04	0.52 ± 0.06	0.735

Values are mean ± SD. *LVEDD* Left ventricle end-diastolic diameter, *LVEDV* Left ventricle end-diastolic volume, *LVESD* Left ventricle end-systolic diameter, *LVESD* Left ventricle end-systolic volume, *FS* Fractional shortening, *EF* Ejection fraction, *SV* stroke volume, *SWTs* septal wall thickness at end-systole, *SWTd* septal wall thickness at end-diastole, *PEP* aortic pre-ejection period, *LVET* Left ventricular ejection time, *RR* inter-beat interval

### Effects of OM versus placebo after 2 months of AR (Table 4)

**Fig. 2:**
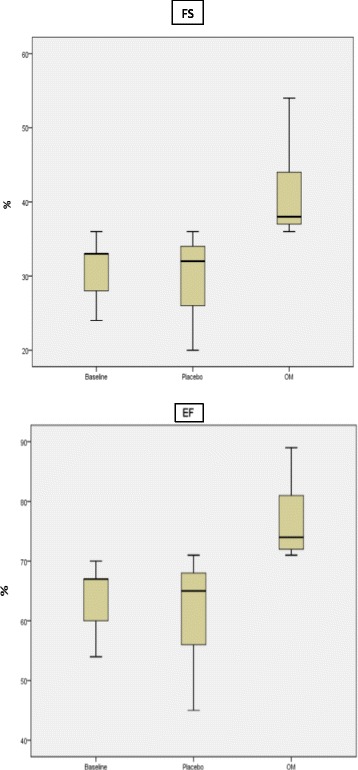
Two-way ANOVA with Bonferroni corrections for multiple comparisons on the effects of OM versus placebo on FS (*p* = 0.014) and EF (*p* = 0.012) after 2 months of AR. Box and Whisker plots before infusion, and after infusion of OM vs. placebo (median: horizontal band within the box, box top and bottom: upper and lower first quartiles, top and bottom whiskers: highest and lowest quartiles; *n* = 12)

Two-way ANOVA with Bonferroni corrections for multiple comparisons after 2 months of AR were done on the effects of placebo versus OM. Only FS and EF increased after OM as compared to placebo (*p* = 0.014 and *p* = 0.012, respectively) (Fig. 2). None of the other hemodynamic changes investigated in this study achieved the level of significance in this analysis (none illustrated).

**Table 4:** Two-way ANOVA statistics with Bonferroni correction at base (T1), before injection (T2) and after injection (T3) for all animals (*n* = 12)

	T1	T2	p	T3	*p*
Heart Rate (beats/min)	226 ± 23	228 ± 21	0.890	208 ± 28	0.113
Left Atrium (mm)	4.6 ± 0.2	6.2 ± 0.3	0.966	5.5 ± 0.2	0.277
LVEDD (mm)	7.9 ± 0.5	11.1 ± 0.4	0.235	10.1 ± 0.5	0.066
LVESD (mm)	5.5 ± 0.4	7.6 ± 0.3	0.871	6.1 ± 0.4	0.092
FS (%)	31.5 ± 1.4	30.5 ± 1.0	0.144	35.4 ± 1.5	0.014
EF (%)	64.1 ± 2.0	63.9 ± 1.4	0.173	68.8 ± 2.5	0.012
Stroke Volume (ml)	0.34 ± 0.03	0.22 ± 0.18	0.129	0.30 ± 0.04	0.135
Cardiac Output (ml/min)	79.5 ± 9.3	52.0 ± 4.5	0.098	61.6 ± 8.6	0.203
SWTs (mm)	2.1 ± 0.1	2.3 ± 0.2	0.597	2.7 ± 0.2	0.652
SWTd (mm)	1.4 ± 0.1	1.7 ± 0.2	0.439	2.1 ± 0.2	0.040
Systolic time (ms)	126 ± 3.7	134 ± 2.6	0.626	135 ± 2.6	0.293
Diastolic time (ms)	139 ± 8.4	135 ± 8.3	0.341	157 ± 14.5	0.293
PEP (ms)	15.0 ± 1.6	25.4 ± 1.3	0.771	21.8 ± 1.0	0.577
LVET (ms)	110 ± 4.4	109 ± 2.8	0.752	113 ± 3.9	0.180
PEP/LVET	0.13 + 0.02	0.23 + 0.02	0.811	0.19 + 0.01	0.378
Systolic time/RR	0.48 + 0.02	0.50 + 0.02	0.567	0.47 + 0.02	0.063

Values are mean ± SD. *LVEDD* Left ventricle end-diastolic diameter, *LVEDV* Left ventricle end-diastolic volume, *LVESD* Left ventricle end-systolic diameter, *LVESD* Left ventricle end-systolic volume, *FS* Fractional shortening, *EF* Ejection fraction, *SV* stroke volume, *SWTs* septal wall thickness at end-systole, *SWTd* septal wall thickness at end-diastole, *PEP* aortic pre-ejection period, *LVET* Left ventricular ejection time, *RR* inter-beat interval

## Discussion

We investigated the effect of the cardiac myosin activator OM on severe chronic AR in an experimental rat-model. The main findings of our study are that OM decreases volume overload induced by chronic AR. As OM lessened LVEDD and LVESD and increased SWTs, we can assume that OM markedly decreased LV wall stress in the presence of a severe chronic AR. We are not aware of a previous similar placebo-controlled study.

### Effects of OM on cardiac function

The central hemodynamic feature of chronic AR is a combined volume and pressure overload of the LV [4, 23]. The LV responds to volume overload with a series of compensatory mechanisms, including a LV dilatation, an increase in chamber compliance and a combination of eccentric and concentric hypertrophy. The ejection phase indexes of LV systolic function at rest remain normal. However, an enlarged chamber size with the associated increase in wall stress also results in a stimulus for further hypertrophy [24]. Despite the small number (*n* = 4) of sham animals, OM increase EF and FS in sham OM group (*n* = 2).

In our study OM decreased volume overload induced by AR during the whole cardiac cycle, by lowering LVEDD. Moreover, by decreasing LVEDD and LVESD and increasing SWTs, OM decreased wall stress in AR. This is of importance, since an increased wall stress may lead to overt LV systolic dysfunction [25]. Improving cardiac systolic function with a cardiac myosin activator could be favorable to ventricular remodeling [26].

In our study OM decreased the PEP/LVET ratio, a reliable index of LV performance. Acute reduction in afterload in patients with congestive heart failure improves LV systolic performance and decreases the PEP/LVET ratio, while an increase in preload will shorten PEP, prolong LVET and decrease PEP/LVET [27]. We found that despite preload reduction by OM, PEP was shortened and cardiac performance improved. This mechanism could explained the increase in stroke volume [28]. An improvement in cardiac function after infusion of OM in mongrel dogs, where heart failure was achieved by rapid ventricular pacing-induced energy depletion, has also been reported, because OM decreased LV end-diastolic pressure without affecting LV systolic pressure [15].

In contrast to our initial hypothesis, we found that OM did not affect the severity of the aortic leakage since the diastolic time-span remained unchanged, as a result of a reduction in HR. In mitigation, however, excessive prolongation the duration of systole might compromise myocardial blood flow, and thereby aggravate ischemia; even if studies with OM in patients with angina and ischemic cardiomyopathy seem reassuring in this regard [29]. This stands in contrast with inotropic drugs that enhance the risk of ischemia, arrhythmias and death. Hence forth those risks have limited their utility in treating acute and chronic heart failure [30].

Ketamine is a dissociative anesthetic agent that has cardiovascular effect resembling sympathetic nervous system stimulation,increase heart rate and cardiac output [31]. Medetomidine improves muscle relaxation, potentiates anesthetic action of ketamine and compensates the cardiac stimulating effect of ketamine by decreasing heart rate and cardiac output. Dexmedetomidine had no direct myocardial depressant effect in the rat heart in doses that are similar to those encountered under clinical conditions [32]. As animals were all anesthetized at the same regime, the decrease in heart rate observed in the OM group can be attributed to OM. However, we cannot predict if this bradycardic effect of OM had also been highlighted in conscious non-sedated animals.

### Possible differential effects of OM as compared to other inotropic agents in AR

Dobutamine infusion, in patients with chronic aortic regurgitation and depressed LV ejection fraction, decreased LVEDD, LVEDV, LVESD and LVESV, while FS and EF improved [33]. In conscious dogs with heart failure, systemic and pulmonary systolic wall stress remained unchanged while HR, LV systolic pressure and LV dP/dt increased with Dobutamine [34]. Dobutamine also shortened LVET in healthy dogs [35]. On the opposite, in our study OM decreased HR and increased LVET, while others, in conscious dogs with systolic heart failure induced by rapid pacing, reported that OM did not affect LV dP/dt [15]. When a comparable concentration of OM than in our study was administrated in normal humans (400 ng/ml) [36], blood pressure did not change. Thus OM and Dobutamine tend to enhance LV contractility by increasing wall thickening and fractional shortening, but in the presence of unchanged afterload conditions with OM [37], while arterial pressure and total vascular resistance increase with Dobutamine.

Currently available inotropes Dobutamin, Dopamin, Milrinone and Levosimendan have demonstrated pro-arrhythmic effects linked to increased mortality that can limit their clinical utility [38]. Most inotropic agents modify calcium cellular homeostasis. This is important, as intracellular calcium plays an important role in myocardial oxygen demand [39]. The well-known and widely used sympathomimetic drug dobutamine increases calcium channels accessibility [40]. Other medications, such as levosimendan, enhance the sensitivity of troponin-c towards calcium, not at the expense of an increase in intracellular calcium concentration [22]. Levosimendan increases contractility by enhancing cross-bridge formation between actin and myosin [22, 41]. The side effect of these increases in contractility is that they raise also myocardial oxygen consumption which is also pro arrhythmic. The molecules may also alter the expression of genes and promote the apoptosis of myocardial cells elicited by the increased intracellular calcium [39, 41]. OM inhibits non-actin dependent cardiac myosin adenosine triphosphate [16] and does not raise myocardial oxygen consumption [15]. A recent study in anesthetized animals suggested the opposite, namely that OM increased myocardial oxygen consumption [42], and however this was apparently undermined by methodological limitations [43]. These favorable characteristics of OM could prove useful in patients with AR.

### Effects of OM dose on the observed changes

In our study we administrated 1.2 mg/kg/h of OM during 30 min. This was expected to raise plasma concentrations of OM to nearly 400 ng/ml [44]. In rats with heart failure induced by a ligation of the left coronary artery, infusion of OM resulted in comparable increases in FS than in our study, starting at plasma concentrations of approximately 200 ng/ml [44]. Administration of less than 0.48 mg/kg/h of OM yield plasma levels < 160 ng/ml, where no LV functional improvements where observed [36]. In healthy human improvements in EF began at a dose of 0.5 mg/kg/h, while improvements in FS, LVET and SV began at an infusion rate of 0.125 mg/kg/h [36]. No change in orthostatic vital signs was noted in this study [45]. In patients with heart failure [45], LVET increased at OM concentrations > 100 ng/ml, while SV and FS raised at plasma levels > 200 ng/ml. EF increased only beginning concentrations > 300 ng/ml. Supine and standing systolic blood pressure decreased at > 400 and > 500 ng/ml, respectively. Last, in the ATOMIC-AHF study [46], patients with acute heart failure treated with OM disclosed a concentration-dependent reduction in HR at a concentration > 200 ng/ml while blood pressure increased at a concentration > 300 ng/ml, as compared to placebo. There was also a concentration-related decrease in LVESD and increase in LVET. OM concentrations > 400 ng/ml achieved a better dyspnea response. Thus the dose administered in our study seems in the upper range of the concentrations where favorable hemodynamic modifications of OM are clear-cut, without being harmful. Adverse effects of OM consist in an excessive prolongation of systolic ejection time > 110 ms [36]. This was observed with supra therapeutic concentrations of OM (~ 1200 ng/ml) which may induce myocardial ischemia by reducing the time during which diastolic coronary blood flow can occur [46]. A drug overdose in a patient with heart failure, with a predicted concentration of 1750 ng/ml at the time of infusion termination, resulted in chest pain, sweating, hypotension, and ECG changes suggestive of ischemia [45]. It is not known, however, that the angina symptoms, observed at supra-therapeutic concentrations of OM, are related - or not - to the presently recognized ryanodine receptors activating effect of OM [47].

### Study limitations

The tested animal group was small because many rats did not recover from the acute AR procedure. Another striking limitation of our study resides in the fact that we could not achieve ventricular blood pressure measurement during the study. As such, our assessment of ventricular loading conditions remains incomplete. Several studies suggest however that systemic blood pressure is not affected at the OM concentrations we achieved in our study [36, 37]. Our study did also not assess whether OM has dose-dependent hemodynamic effects in our model of AR. Lower doses of OM may still exert favorable hemodynamic effects, while even further reducing the risk of excessive prolongations in LVET. This will require additional studies. Moreover, as already discussed, a direct comparison of the effects of different inotropic agents in AR-related heart failure would also provide further insights in the differential hemodynamic effects of OM, as compared to other inotropic agents. Last, the effects of OM on animals who might otherwise not survive the decompensation period after an acute AR should be also studied. This is a very poorly tolerated condition in humans [48], which could benefit from further studies on the best hemodynamic support while awaiting cardiac surgery [48]. Last, the sham group in our study comprised only 2 animals in the OM group and 2 animals in the placebo group.

Another limitation of the study, the possible fistula with left atrium created during aortic valve leaflets puncture, this fistula can explain the decrease of stroke volume and cardiac output after the creation of the aortic regurgitation.

## Conclusion

The present placebo-controlled study shows improvements in cardiac function after infusion of OM. Our investigations demonstrate these effects for the first time in the rat-model with chronic severe AR. We observed a decrease in volume overload and an increase in cardiac output and wall thickness. Moreover OM enhanced EF and FS, while coincidently lowering the HR and wall stress. On the other hand, OM did not affect the duration of diastole and the severity of AR.

## Abbreviations

AR: Aortic regurgitation
ATP: Adenosin triphosphate
EF: Ejection fraction
FS: Fractional shortening
HR: Heart rate
LV: Left ventricle
LVEDD: Left ventricle end-diastolic diameter
LVESD: Left ventricle end-systolic diameter
LVET: Left ventricular ejection time
OM: Omecamtiv-mecarbil
PEP: Pre-ejection period
Pi: Hydrolazed phosphate
RR: Inter-beat interval
SV: Stroke volume
